# A First Case Report of DiGeorge Syndrome from Ethiopia Highlights Challenges in Identifying and Treating Children with Primary T-Cell Deficiencies in Low Resource Settings

**DOI:** 10.1155/2020/8157212

**Published:** 2020-02-26

**Authors:** Tinsae Alemayehu, Solomie Jebessa Deribessa

**Affiliations:** ^1^American Medical Center, Specialty Clinic for Infectious Diseases and Travel Medicine, Addis Ababa, Ethiopia; ^2^Department of Pediatrics and Child Health, St. Paul's Hospital and Millennium Medical College, Addis Ababa, Ethiopia

## Abstract

**Background:**

Cellular primary immunodeficiencies are rarely reported from Africa. DiGeorge syndrome is a commonly recognized form of a congenital T-cell deficiency. The disorder is characterized by hypoplastic or aplastic thymus, hypocalcemia, recurrent infections, and other associated congenital defects. *Case Report*. We report an eleven-month-old infant presenting with recurrent chest and diarrheal infections, failure to thrive, lymphopenia, hypocalcemia, and hypoplastic thymus on imaging. A diagnosis of DiGeorge syndrome was confirmed after determining very low CD3 and CD4 levels.

**Conclusions:**

We describe the first case report of an Ethiopian child with a congenital T-cell immunodeficiency. We have outlined essentials for diagnosis and management of cellular primary immunodeficiency disorders in low resource settings.

## 1. Introduction

Primary immune-deficiencies (PIDs) are rarely reported among African children. The bulk of available data is derived from patient registries in North African countries and South Africa [1, 2]. Excluding those countries, the presence of only a handful of pediatric case reports (from Kenya, Ethiopia, Zambia, and South Africa) shows the scarcity of knowledge concerning PIDs in the majority of African countries [3–6].

Primary immunodeficiencies are classified into nine categories. The commonest disorders are antibody deficiencies which account for half of all forms of PIDs, while congenital T-cell deficiencies account for 20–30% [7].

DiGeorge syndrome is a well-described congenital T-cell defect characterized by a lack of thymic and pharyngeal arch development. It predisposes affected infants for repeated bacterial, fungal, and protozoal infections from early infancy onwards. Affected patients rarely survive past their first year [6]. The disorder has never been reported from Ethiopian patients.

We report an eleven month male infant with severe immunodeficiency due to DiGeorge syndrome. We also highlight principles of evaluating children with primary cellular immunodeficiencies and describe difficulties faced to diagnose and treat him in low-income countries like Ethiopia.

## 2. Case Presentation

An 11-month-old boy from Addis Ababa, Ethiopia, presented with difficulty of breathing, cough, frequent interruptions of breast feeding, and high-grade fever of 3 days to the pediatric department of Tikur Anbessa Specialized Hospital, Addis Ababa, Ethiopia.

He was born at 42 weeks of gestational age (birth weight: 3300 grams) after an uneventful pregnancy. He is an only child to nonconsanguineous parents. His past history was notable for being treated three times (out-patient and in-patient) for pneumonia since 2 months of age. He had multiple episodes of acute diarrhea. He had received all vaccines according to the national schedule, and parents did not notice any postvaccine reactions. There was no history of recurrent infections in early life among immediate family members.

On physical examination, he was acutely sick-looking with marked respiratory distress. His temperature was 38.7°C, respiratory rate 72 per minute, pulse rate 145 per minute, and his saturation of oxygen at room air measured 77%. He was underweight (5 kg) and stunted (59 cm) with an age-appropriate head circumference (47 cm). He had pink conjunctivae with nonicteric sclerae. He had no cleft lip/palate with normal ear and chin sizes. There were no palpable lymph node enlargements.

He had flaring of ala nasi, intercostal and subcostal retractions, bilateral bronchial breath sounds, and fine crepitations in the right lung field. There were normal heart sounds with no murmur or gallop. He had no organomegaly or signs of fluid collection on abdominal examination. There were no active lesions or edema. No skeletal deformity or focal neurologic deficit was noted.

**Figure 1:**
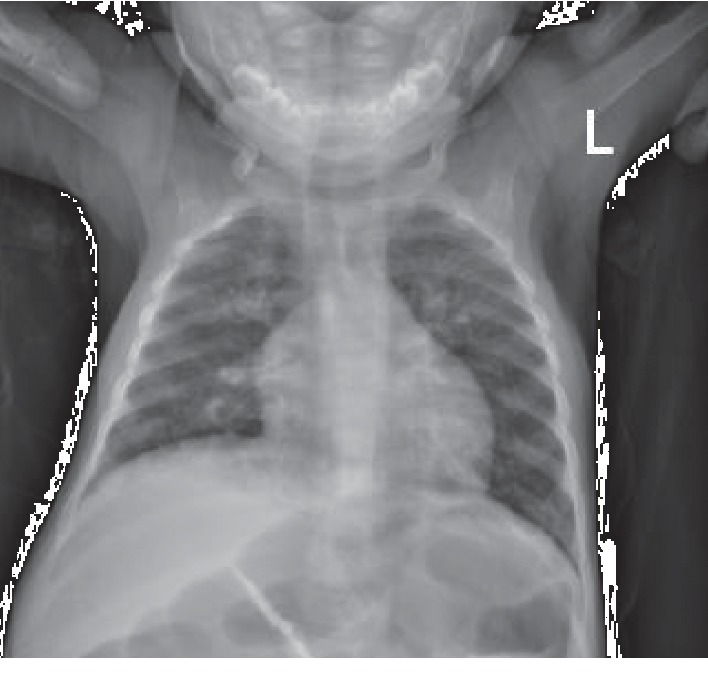
Chest x-ray of the infant.

His complete blood count revealed a white blood cell count of 9710/mm^3^, neutrophils of 5900/mm^3^, lymphocytes of 1390/mm^3^ (definition for lymphopenia for ages 7–24 months: less than 3400/mm^3^), and eosinophils of 320/mm^3^ and monocytes of 1880/mm^3^. A chest x-ray showed ground glass opacities over right upper and lower lobes and almost no thymic shadow suggesting hypoplastic thymus for his for age (Figure 1). The findings were confirmed by a chest CT scan.

Serum ionized calcium level was 1.2 mg/dl (normal: 2.1–2.5 mg/dl), while his serum parathyroid level was slightly elevated at 77.92 pg/ml (normal 14–72 pg/ml). A diagnosis of DiGeorge syndrome was confirmed after doing T-cell-specific CD4 measurements. His CD3 measured 323/mm^3^ (normal for age: 2300–4800/mm^3^) while his CD4 was 86/mm^3^ (normal for age: 1500–3500/mm^3^). He was not seroreactive for HIV. He had a normal echocardiographic study. His serum antinuclear antibody (ANA) test was negative. Further autoimmune panel testing and detection of a deletion in chromosome 22 was not possible in the hospital.

He was managed by intravenous antibiotics, following which he was placed on cotrimoxazole prophylaxis. He began follow-up at the pediatric infectious diseases' clinic of the hospital, while an arrangement for referral for thymic transplantation with or without bone marrow transplantation (both of which are unavailable in Ethiopia) was made.

## 3. Discussion

Very few reports of primary immunodeficiencies are made from Africa. Based on a 2014 global survey, only 1.9% of identified patients with such disorders were from Africa, while the continent is home to 16.7% of the world's population [8].

T-cell defects are identified among 30% of all primary immunodeficiencies. DiGeorge syndrome is the commonly recognized member while severe combined immunodeficiency (SCID) is the most severe [9].

Red flags showing a likely T-cell immune defect include early age at onset (younger than 6 months), protracted and recurrent opportunistic infections (viruses, *pneumocystis jirovecii*, mycobacteria, *candida*, etc), and adverse reactions after live vaccines [10]. DiGeorge syndrome (DGS) is characterized by a defect in the development of the 3rd and 4th pharyngeal pouches. It presents with thymic hypoplasia (partial DGS with normal growth and development) or aplasia (complete DGS), hypocalcemia, and parathyroid hypoplasia [11]. Newborns may have esophageal atresia as well as congenital cardiac defects like conotruncal anomalies. It is attributed to a deletion of chromosome 22q1.2 [12].

Though affected patients usually exhibit a dysmorphism notable for a bifid uvula, short philtrum of the upper lip, mandibular hypoplasia, low-set ears, hypertelorism, and an antimongoloid slanting of the eyes, these features are not often seen in children of African descent like our patient [6].

Our patient presented with repeated respiratory and diarrheal infections. He exhibited many of the characteristic features of DGS: hypoplastic thymus, hypocalcemia, elevated parathyroid levels, lymphopenia, and markedly low CD3 and CD4 levels for age. Most cases of DGS are sporadic and hence have no familial history of recurrent infections. Confirmatory genetic studies could not be conducted for our patient as they were not available.

Children with primary T-cell deficiencies and their immunocompetent household contacts should not receive live attenuated vaccines, viral and bacterial, except for pneumococcal and *Hemophilus influenzae* type b vaccines [13]. Patients may also require prophylactic trimethoprim-sulfamethoxazole till a definitive treatment is given [10, 14].

In conclusion, we report an 11-month-old infant with DiGeorge syndrome. This is the first case report of an Ethiopian child with a congenital T-cell immunodeficiency. Though complete diagnostics and treatment for primary immunodeficiencies are lacking in Ethiopia, this case report will help create awareness among clinicians on the presentation of DiGeorge syndrome. Optimizing immunizations, infection control while administering antimicrobial prophylaxis are the main components of management of children with congenital T-cell deficiencies in low resource settings, while we strive for stem cell transplantations.

## References

[1] Barbouche M.-R., Galal N., Ben-Mustapha I. (2011). Primary immunodeficiencies in highly consanguineous North African populations. *Annals of the New York Academy of Sciences*.

[2] Naidoo R., Ungerer L., Cooper M., Pienaar S., Eley B. S. (2011). Primary immunodeficiencies: a 27-year review at a tertiary paediatric hospital in cape town, South Africa. *Journal of Clinical Immunology*.

[3] Alemayehu T., Tefera E. (2017). Dilated cardiomyopathy in a child with hyper-immunoglobulin E syndrome. *East African Medical Journal*.

[4] Chipeta J., Banda J., Mbinga M., Wa-Somwe S. (2009). Absent uvula and thrombocytopenia in an African infant with Job’s syndrome: case report and a review of literature. *Journal of Infectious Diseases & Immune*.

[5] Trikamjee T., Levin M. (2016). A rare case of hyper IgE syndrome. *Current Allergy & Clinical Immunology*.

[6] Walong E., Rogena E., Sabai D. (2014). Primary immunodeficiency diagnosed at autopsy: a case report. *BMC Research Notes*.

[7] Bousfiha A., Jeddane L., Picard C. (2018). The 2017 IUIS Phenotypic classification for primary immunodeficiencies. *Journal of Clinical Immunology*.

[8] Modell V., Knaus M., Modell F., Roifman C., Orange J., Notarangelo L. D. (2014). Global overview of primary immunodeficiencies: a report from Jeffrey Modell Centers worldwide focused on diagnosis, treatment, and discovery. *Immunologic Research*.

[9] Cooper M. A., Pommering T. L., Korányi K. (2003). Primary immunodeficiencies. *American Family Physician*.

[10] Eley B., Esser M. (2014). Investigation and management of primary immunodeficiency in South African children. *South African Medical Journal*.

[11] Digilio M., Marino B., Capolino R., Dallapiccola B. (2005). Clinical manifestations of deletion 22q11.2 syndrome (DiGeorge/Velo-Cardio-Facial syndrome). *Images in Paediatric Cardiology*.

[12] McDonald-McGinn D. M., Minugh-Purvis N., Kirschner R. E. (2005). The 22q11.2 deletion in African-American patients: an underdiagnosed population?. *American Journal of Medical Genetics Part A*.

[13] Shearer W. T., Fleisher T. A., Buckley R. H. (2014). Recommendations for live viral and bacterial vaccines in immunodeficient patients and their close contacts. *Journal of Allergy and Clinical Immunology*.

[14] Rubin L. G., Levin M. J., Ljungman P. (2014). 2013 IDSA clinical practice guideline for vaccination of the immunocompromised host. *Clinical Infectious Diseases*.

